# Breast Cancer Suspicion in a Transgender Male-to-Female Patient on Hormone Replacement Therapy Presenting with Right Breast Mass: Breast Cancer Risk Assessment and Presentation of a Rare Lesion

**DOI:** 10.1155/2017/5172072

**Published:** 2017-03-21

**Authors:** Krystina Tongson, Victoria Konovalova, Naveen Dhawan, Steffi Sharma, Jaya Bahl, Mohammad Masri

**Affiliations:** ^1^Department of General Surgery, Larkin Community Hospital, South Miami, FL, USA; ^2^Division of Health Sciences, Nova Southeastern University, Davie, FL, USA; ^3^Kasturba Medical College, Manipal, India; ^4^Florida International University (FIU), Miami, FL, USA

## Abstract

There has been an increasing use of hormonal therapy among male-to-female (MtF) transgender individuals. This long-term hormone replacement therapy (HRT) renders MtF individuals a unique patient subgroup in terms of breast cancer risk. This case describes a MtF transgender who presented with a breast lesion concerning for malignancy following hormonal replacement therapy. The patient additionally had a strong family history of breast cancer. Final pathology revealed lobular hyperplasia in the setting of gynecomastia and pseudoangiomatous stromal hyperplasia (PASH). Both pathology findings are rare in biological females, let alone in the setting of hormone replacement therapy in a MtF individual. While the number of reported cases of suspicious breast lesions in this population remains scarce, it presents both a diagnostic and therapeutic challenge due to the nature of the treatment course and the lack of research in this recently growing subgroup of patients.

## 1. Introduction

Male-to-female (MtF) transgender individuals are defined as those born with a male genotype and yet who psychosocially identify as female [1]. Recent reports demonstrate that the prevalence of gender dysphoria for MtF transgender individuals is estimated to be 1 in 10 to 1 in 25,000 [2]. To maintain secondary female characteristics, MtF transgender individuals may elect to undergo hormone replacement therapy [1, 3]. It has been well-established that HRT is a risk factor for development of breast cancer; although breast cancer in men is rare, the relative risk from exposure of exogenous estrogen in the MtF transgender population has not yet been defined [4, 5]. Furthermore, the combined influence of breast cancer risk factors, such as family history and BRCA gene mutations, in the transgender population has yet to be described in current literature. Overall, the occurrence and attributes of breast cancer onset among transgender patients have not been adequately evaluated.

We report a case of a MtF transgender with a strong family history of breast cancer who underwent HRT and subsequently developed a breast lesion with histological findings compatible with lobular hyperplasia with focal areas of pseudoangiomatous stromal hyperplasia (PASH). This is one of the few case reports in the literature that describes a case of a MtF transgender with the concern of breast cancer following HRT. It is also one of the few cases reported of lobular hyperplasia in the setting of gynecomastia and PASH in a transgender patient. Additionally, this case highlights the need for screening recommendations in high-risk MtF transgender patients. This is particularly important considering the growing number of transgender patients in the US and around the world that undergo hormone replacement therapy.

## 2. Case Presentation

A 38-year-old MtF transgender patient presented to our hospital with a right-sided breast mass in August 2016. She was receiving HRT with estrogen and progesterone since June 2015. During this time, the patient's treatment was halted from October to December 2015 due to an elevation in hormonal levels. Six months after the initiation of HRT, the patient had discovered a lump in her right breast in the 9 o'clock position. On physical exam, the mass was fixed and firm and the patient reported an increase in size. No additional masses were appreciated in either breast or axillae. While reviewing the patient's breast cancer risk, it was noted that the patient has two sisters with breast cancer, both of whom were diagnosed before the age of forty years old; one had passed away from breast cancer and the other is in remission.

She denied noting any masses in the axilla, weight loss, or night sweats. Other than the lump in the right breast, the physical examination was unremarkable. Her past medical history included gender dysphoria, anxiety, bipolar disorder, and essential hypertension. She had no surgeries in the past.

**Figure 1:**
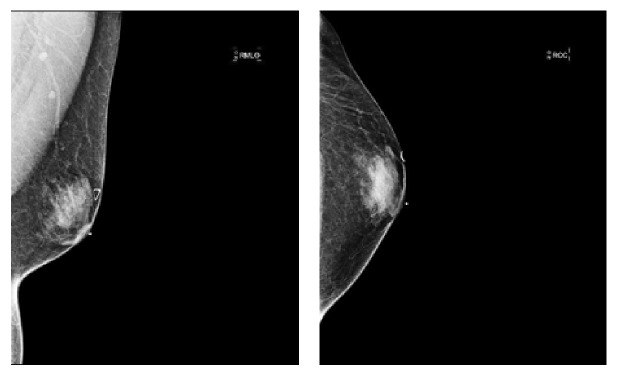
Mammogram of right breast.

**Figure 2:**
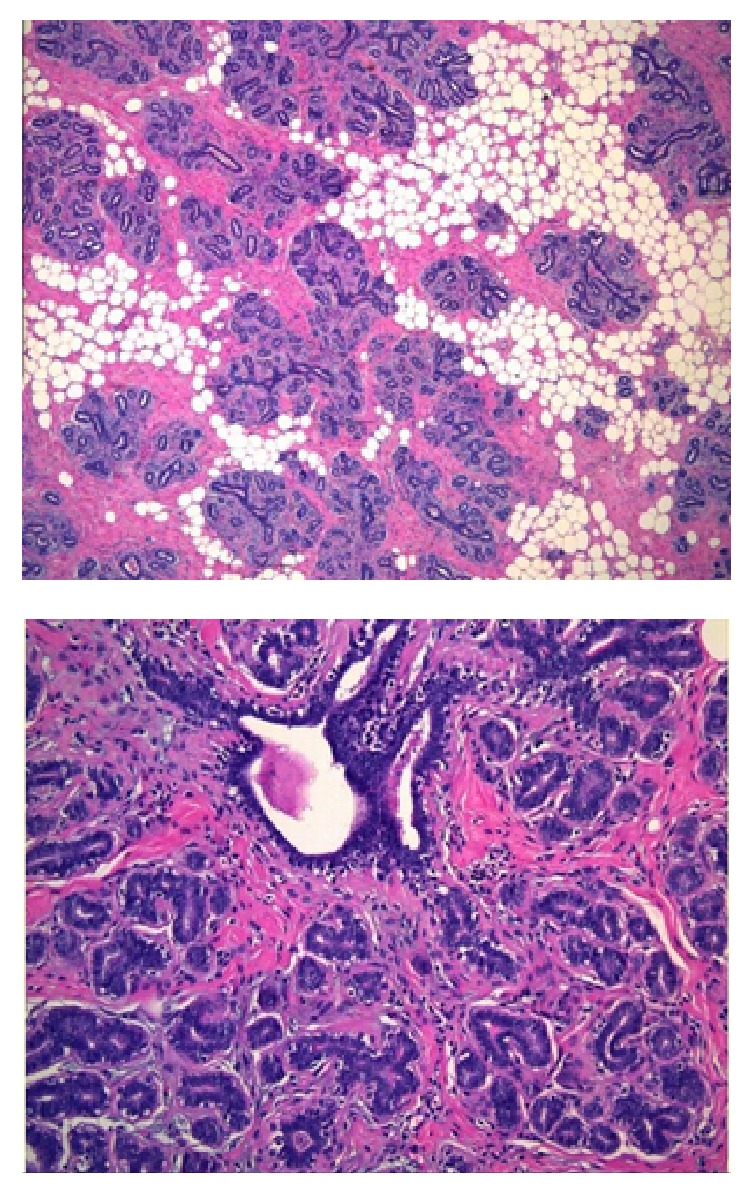
Pathology report: lobular hyperplasia.

**Figure 3:**
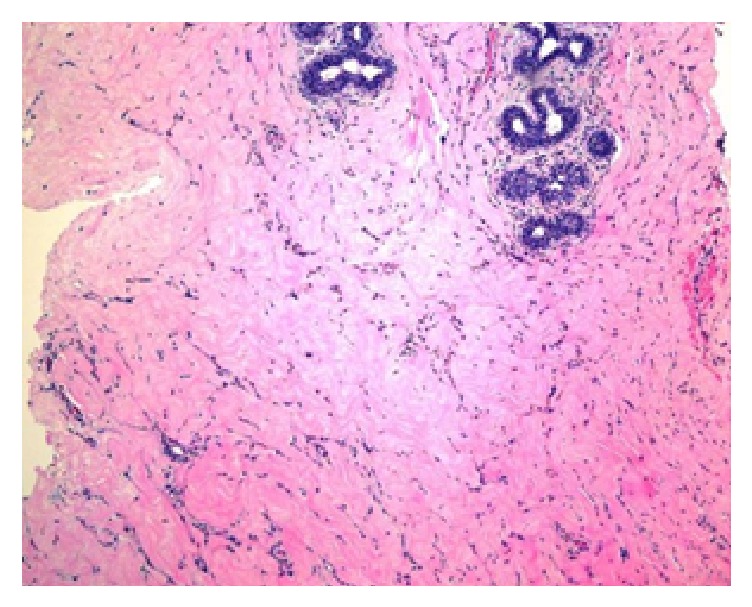
Pathology report: PASH-like changes.

Imaging (bone density scan, mammogram, and ultrasound) was performed for further assessment. A bone density scan of the lumbar spine and the left hip revealed normal bone density without any notable risk of fracture. Mammography revealed BIRAD 2 classification of right-sided breast mass and bilateral gynecomastia (Figure 1). An ultrasound of the right breast showed no focal lesions, nodules, or masses in the right breast. Dense mammary parenchyma was noted, particularly in the upper outer quadrants, consistent with gynecomastia. While imaging did not suggest malignancy, the patient's strong family history of breast cancer in conjunction with her history of undergoing HRT raised a high index of suspicion. The patient also desired a definitive diagnosis. Our team therefore decided to perform a lumpectomy of the right breast mass. Pathology revealed lobular hyperplasia with focal pseudoangiomatous stromal hyperplasia (PASH), a rare benign entity (Figures 2 and 3). Lobular hyperplasia in the setting of gynecomastia in a male patient is especially rare.

## 3. Discussion

In some countries, 1 in 11,900–12,900 males and 1 in 30,400–33,800 females are estimated to identify themselves as transgender individuals [6, 7]. Male-to-female (MtF) transgender individuals have a male genotype from birth but have a self-perception of being female. Many of these individuals undergo hormone therapy to sustain secondary female traits as part of their sex change [1, 3]. Many MtF transgender patients prefer to use nonhormonal or hormonal treatments to develop female secondary sexual characteristics, including gender-reassignment surgery [1]. Of concern is the fact that the doses of estrogen that are consumed by this population are higher than those prescribed to hypogonadal women as substitutive therapy and the higher doses are continued beyond the average female menopausal age [1].

While hormone therapy is known to be a risk factor for breast cancer in women, the risk in MtF transgender individuals has not been established [4, 5]. Importantly, there is a lack of adequate data on transgender patients due to a paucity of reported cases in the literature regarding this distinct patient population. Further compounding this problem are generalized notions patients hold regarding breast cancer; the fact that some MtF transgender patients reportedly believe that breast cancer only occurs in females warrants greater efforts on the part of clinicians to educate patients [8].

Identifiable risk factors for breast cancer in men include BRCA gene mutations, family history, androgen deficiency, and estrogen exposure [9]. The first documented case of breast cancer in a MtF transsexual with a pathogenic BRCA2 mutation was described by Corman et al. [10]. In our case, the surgical service was consulted on a transgender MtF patient with a strong family history of breast cancer who underwent hormone replacement therapy and presented with a breast lesion. While our index of suspicion was high for breast cancer given the patient's history, the biopsy results ultimately revealed a benign but rare lesion. Our team was faced with the question of whether to proceed with a less invasive core needle biopsy or a more aggressive biopsy, such as excisional biopsy, for definitive tissue diagnosis. This experience has now prompted us to be prepared for this patient population and to consider the prognostic and diagnostic challenges.

Ultimately, our team resected the lump despite the negative image results, due to our high index of suspicion and discordance of imaging with physical exam findings. The final pathology of the specimen demonstrated lobular hyperplasia with focal areas of pseudoangiomatous stromal hyperplasia (PASH). Lobular hyperplasia is a benign and rare lesion that is uncommon in gynecomastia [11, 12]. PASH is a rare benign lesion typified by stromal myofibroblastic proliferation that is more common in premenopausal women; only 100–200 cases have been described in the literature [13, 14].

Currently, primary care physicians are recommended to offer breast cancer screening to all transgender individuals in accordance with national guidelines [15]. Yet, the use of mammography in transgender individuals has not been adequately studied. Importantly, one retrospective study of transgender individuals at a community health center in Massachusetts showed that transgender individuals were not as likely as cisgender patients to follow guidelines of mammography screening [16]. Maglione et al. analyzed 8 existing cases and described two original cases (65-year-old and a 55-year-old that had a family history of breast cancer) of MtF transgender individuals who developed breast cancer, reporting that breast cancer occurs at a younger age and is more often ER (estrogen receptor) positive compared to males [5]. Their experience using mammography with the two patients bolsters the notion that mammography should be indicated for MtF patients, particularly those with specific risk factors (use of hormonal therapy, exposure to radiation throughout life, BRCA gene mutations, Klinefelter syndrome, and family history) [5].

In studies conducted on men with gynecomastia, it has been suggested that, in men that were found to have gynecomastia on incidental chest CT, mammography within 8 months of the CT will most likely not show malignancy (assuming no other suspicious findings on physical exam) [17]. Thus, per one retrospective analysis of 62 men, cross-sectional imaging is sufficient in men with signs of gynecomastia; mammography is not recommended [17].

## 4. Conclusion

This case highlights the importance of recognizing the possibilities of breast cancer risk for male-to-female transgender patients who undergo HRT, as well as those patients with strong family history of breast cancer. Although rare, the documented cases of invasive breast cancer in MtF transgender have been found at very advanced stages. Some MtF transgender individuals reportedly hold the belief that breast cancer occurs exclusively in females [8]. This stresses the need for clinicians to educate transgender patients about hormone therapy.

The occurrence and attributes of breast cancer onset in transgender individuals have not been adequately studied. This is due to the rarity of the disease and patient population. There appears to be a link between BRCA 1 and BRCA 2 genes and an increased incidence of breast cancer in MtF transgender individuals. The link between hormonal therapy and breast cancer risk among the MtF transgender population is controversial. The two largest studies, a cohort study in Europe and a retrospective study conducted in North America, suggest that there is no significant association. Still, clinicians should continue to maintain a high index of suspicion and exercise awareness of the use of hormonal therapy and genetic factors that predispose this population to the risks of breast cancer. Mammography is currently recommended for MtF transgender individuals, although no major studies have investigated its specific utility in screening for this population. The use of a clinical decision tool could translate into improved preventive care for this growing and often underserved patient population.
